# Identification of co-diagnostic effect genes for aortic dissection and metabolic syndrome by multiple machine learning algorithms

**DOI:** 10.1038/s41598-023-41017-4

**Published:** 2023-09-08

**Authors:** Yang Zhang, Jinwei Li, Lihua Chen, Rui Liang, Quan Liu, Zhiyi Wang

**Affiliations:** 1 Kunming Medical University, Kunming, 650000 Yunnan China; 2 Department of Vascular Surgery, Fuwai Yunnan Cardiovascular Hospital, Affiliated Cardiovascular Hospital of Kunming Medical University, Kunming, 650000 Yunnan China; 3 Department of Neurosurgery, The Fourth Affiliated Hospital of Guangxi Medical University, Liuzhou, 545000 Guangxi China; 4 Department of Neurosurgery, West China Hospital, Sichuan University, Chengdu, Sichuan, 610000 China; 5 Department of Cardiology, the Second Affiliated Hospital of Chongqing Medical University, Chongqing, 400010 China; 6 College of Bioengineering, Chongqing University, Chongqing, 400030 China; 7 Vascular Surgery, the First Affiliated Hospital of Dali University, Dali, 671000 China

**Keywords:** Biomarkers, Cardiology, Diseases, Molecular medicine

## Abstract

Aortic dissection (AD) is a life-threatening condition in which the inner layer of the aorta tears. It has been reported that metabolic syndrome (MS) has a close linkage with aortic dissection. However, the inter-relational mechanisms between them were still unclear. This article explored the hub gene signatures and potential molecular mechanisms in AD and MS. We obtained five bulk RNA-seq datasets of AD, one single cell RNA-seq (scRNA-seq) dataset of ascending thoracic aortic aneurysm (ATAA), and one bulk RNA-seq dataset of MS from the gene expression omnibus (GEO) database. Identification of differentially expressed genes (DEGs) and key modules via weighted gene co-expression network analysis (WGCNA), functional enrichment analysis, and machine learning algorithms (Random Forest and LASSO regression) were used to identify hub genes for diagnosing AD with MS. XGBoost further improved the diagnostic performance of the model. The receiver operating characteristic (ROC) and precision-recall (PR) curves were developed to assess the diagnostic value. Then, immune cell infiltration and metabolism-associated pathways analyses were created to investigate immune cell and metabolism-associated pathway dysregulation in AD and MS. Finally, the scRNA-seq dataset was performed to confirm the expression levels of identified hub genes. 406 common DEGs were identified between the merged AD and MS datasets. Functional enrichment analysis revealed these DEGs were enriched for applicable terms of metabolism, cellular processes, organismal systems, and human diseases. Besides, the positively related key modules of AD and MS were mainly enriched in transcription factor binding and inflammatory response. In contrast, the negatively related modules were significantly associated with adaptive immune response and regulation of nuclease activity. Through machine learning, nine genes with common diagnostic effects were found in AD and MS, including *MAD2L2, IMP4, PRPF4, CHSY1, SLC20A1, SLC9A1, TIPRL, DPYD,* and *MAPKAPK2*. In the training set, the AUC of the hub gene on RP and RR curves was 1. In the AD verification set, the AUC of the Hub gene on RP and RR curves were 0.946 and 0.955, respectively. In the MS set, the AUC of the Hub gene on RP and RR curves were 0.978 and 0.98, respectively. scRNA-seq analysis revealed that the *SLC20A1* was found to be relevant in fatty acid metabolic pathways and expressed in endothelial cells. Our study revealed the common pathogenesis of AD and MS. These common pathways and hub genes might provide new ideas for further mechanism research.

## Introduction

Aortic dissection is an acute condition where blood enters the medial layer of the aorta and causes sudden death^1^. The most life-threatening vascular disease is Type A aortic dissection (ATAAD) with dissected ascending aorta^2,3^, with mortality as high as 1% per hour^4^. Different types of pathological conditions can cause aortic dissection. Atherosclerosis, cystic medial necrosis, and other degenerative processes can affect the aorta^5^. The aortic wall consists of a highly dynamic cell population and extracellular matrix (ECM) that performs complex biomechanical functions to provide appropriate compliance and sufficient strength to cope with hemodynamic changes. Dysregulation of these components leads to progressive depletion of smooth muscle cells (SMCS), disruption of the ECM, and inflammation leading to aortic aneurysms, dissection, and rupture^6^. Although multiple cellular mechanism studies have been analyzed in AD, there are still fewer molecular tests for non-invasive.

Metabolic syndrome (MS) consists of several interrelated physiological, biochemical, clinical, and metabolic factors that increase the risk of cardiovascular disease and T2DM as well as all-cause mortality^7^. Its clinical recognition is based on insulin resistance accompanied by elevated plasma insulin levels, visceral adiposity, atherogenic dyslipidemia (including high triglycerides and LDL cholesterol as well as reduced HDL cholesterol), endothelial dysfunction, elevated blood pressure, and a hypercoagulable state. Today, it is widely accepted that a pro-inflammatory state is a component of multiple sclerosis^8^.

It is well recognized that MS can accelerate cardiovascular disease proceeding. AD and MS have many overlapping pathogenic factors and pathogenesis, including SMCs and endothelial dysfunction, local and systemic immune processes, inflammatory cytokines/chemokines such as serum TNF-α, vascular endothelial growth factor, and related enzymes and signaling pathways^6,8–10^. However, people know little about the common features of them based on gene regulation mechanisms.

Recent advances in second-generation sequencing and single-cell sequencing technologies have made it possible to explore the common pathogenesis of disease-disease interactions at the genetic level^11,12^. This study aims to identify hub genes related to the pathogenesis of AD with MS. We analyzed six gene expression data sets (GSE52093, GSE98770, GSE147026, GSE153434, and GSE190635 datasets of AD and GSE98895 dataset of MS) downloaded from the GEO database. Comprehensive bioinformatics and enrichment analysis were used to determine the common DEGs and their functions for AD and MS. In addition, a weighted gene co-expression network was constructed to analyze gene modules and identify hub genes using machine learning algorithms. Specifically, we identified 9 essential hub genes, and we further explored and validated the diagnostic efficiency of these genes. After that, immune cell infiltration and metabolism-associated pathways analyses were created to investigate immune cell and metabolism-associated pathway dysregulation of these genes in AD with MS. In the end, we further analyzed the transcription profiles of these genes and verified their expression levels in a scRNA-seq dataset (GSE213740) of AD. The hub genes identified here between AD and MS are expected to provide new insights into the biological mechanisms underlying both diseases.

## Materials and methods

### Datasets and data preprocessing

**Figure 1:**
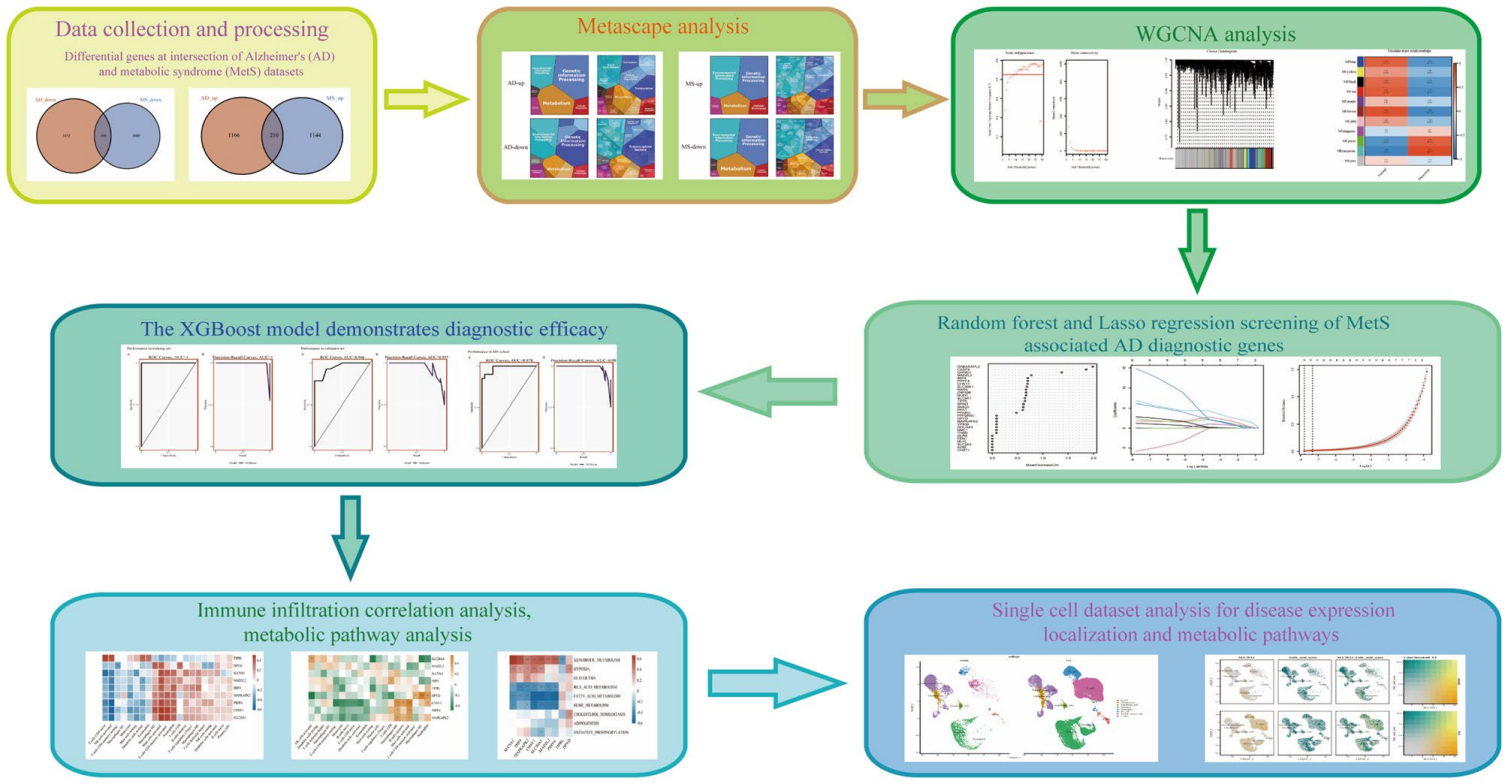
Study flowchart.

Our technical route was illustrated in Fig. 1. We obtained the GEO (https://www.ncbi.nlm.nih.gov/geo/) database's original gene expression profile data and clinical information^13^. We searched for related gene expression datasets using type A aortic dissection and metabolic syndrome as keywords. Finally, bulk RNA-seq datasets numbered GSE52093, GSE98770, GSE147026, GSE153434, GSE190635, and GSE98895 were downloaded from the GEO database using the R package “GEOquery”^14^.

The GSE52093 dataset was used on the GPL10558 platform. The dataset contained 12 samples, including 7 dissected ascending aorta samples (n = 7) and 5 normal ascending aorta samples (n = 5). The GSE98770 dataset was used on the GPL14550 platform. This dataset contained 11 samples, including 6 intima-media layers of dissected ascending aorta samples obtained intraoperatively from acute type A aortic dissection (ATAAD) patients without the familial thoracic aortic disease (n = 6) and 5 of non-dissected ascending aorta samples obtained from transplant donors (n = 5). The GSE147026 dataset was used on the GPL24676 platform. The dataset contained 8 samples, including 4 aortic media tissue samples obtained from aortic dissection (AD) patients (n = 4) and 4 normal donor samples (n = 4). Besides, the GSE153434 dataset based on GPL20795 and the GSE190635 dataset based on GPL570 were regarded as external validation sets. The GSE153434 dataset contained 20 samples, including 10 cases of type A aortic dissection (TAAD) patients as the experimental group (n = 10) and 10 cases of normal ascending aortic tissue samples as the control group (n = 10). The GSE190635 dataset contained 8 samples, including 4 cases of male acute aortic dissection patients as the experimental group (n = 4) and 4 cases of healthy male samples as the control group (n = 4). Finally, the GSE98895 dataset contained 40 samples, including 20 patients with metabolic syndrome (MS) (n = 20) and 20 healthy subjects (n = 20).

To eliminate batch effects across all samples, the Rank-In (http://www.baddcao.net/rank-in/index.html) online platform was utilized and presented the results of batch effect elimination through a PCA graph^15^. The Rank-In algorithm is basically divided into the following three steps: (1) in all the data sets that need to be integrated, the pairs of genes according to the expression value and signal value rank from low to high, the lowest is 1, the highest is 100, and the middle is expressed by percentile (rounding down), which is called internal rank (Internal ranking). (2) for each gene in each expression profile, the gene expression weight value (weight) is calculated by using the gene expression value and internal rank value, and the weighted rank matrix (Weighted ranking matrix) can be obtained by the product of gene expression matrix and gene weight. (3) the corrected matrix of the gene is obtained by singular value decomposition (SVD). Subsequently, the aortic dissection datasets were combined, the common genes from the five datasets were collected, and a new gene expression profile for all samples was formed.

### Identification and enrichment analysis of DEGs

The differentially expressed genes (DEGs) in the AD combined dataset and GSE98895 dataset were determined by comparing gene expression profiles between the diseased and control groups using the 'limma' package. The LogFC ≠ 0 and adj. P < 0.05 were considered to indicate statistical significance. Venn diagram obtained the common DEGs. Kyoto Encyclopedia of Genes and Genomes (KEGG) Pathway was a database that stored information on gene pathways in different species^16^. The Metascape was a gene enrichment tool website. Heat maps were used to show the enrichment of up-regulated and down-regulated genes co-expressed by AD and MS, respectively.

### Weighted gene co-expression network analysis between AD, MS, and normal tissues

Co-expressed gene modules were found using the WGCNA algorithm^17^. Module eigengene values were determined using the "moduleEigengenees" algorithm and gene expression profiles were summarized for specific modules. Hierarchical clustering and Spearman correlation analysis were performed between the Eigengene value of the module and the clinical characteristics of the samples. P < 0.05 and correlation coefficient (r) > 0.25 were statistically significant^18^, and these modules were identified as aortic dissection- or metabolic syndrome-related key modules and selected for further analysis. To analyze the biological functions and pathways involved in these positively or negatively related key modules.

### Identification of hub genes via machine learning

Two machine learning algorithms were adopted to further filter candidate genes for diagnosing AD with MS. RF is a suitable method that has the advantage of not restricting variable conditions, has better accuracy, sensitivity, and specificity, can be used to predict continuous variables, and provides predictions without significant variation^19^. LASSO is a regression method for selecting variables to improve prediction accuracy and a regression technique for variable selection and regularization to improve the prediction accuracy and comprehensibility of statistical models^20^. 'RandomForest'^21^ and 'glmnet'^22^ R packages were used to perform RF and LASSO regression analysis. RF and LASSO intersection genes were considered hub genes in diagnosing AD with MS. We used the up-regulated and down-regulated genes co-expressed by AD and MS as gene sets. A total of 406 genes were used as input genes and dimension reduction was screened. The AD dataset of GSE52093, GSE98770 and GSE147026 was used as the training set (17 AD patients and 14 normal patients). The diagnostic model of HUB gene was established by using XGBoost algorithm, and verified in AD dataset numbered GSE153434 and GSE190635 (14 AD patients and 14 normal patients) and MS dataset^23^ (20 MS patients and 20 normal patients). ROC and Precision-Recall curves were drawn, and the area under the curve (AUC) was used to evaluate the diagnostic efficacy.

### The correlation between AD and MS in immune cell infiltration compared with normal tissues

A bioinformatics algorithm called "CIBERSORT" was used to evaluate immune cell infiltrations. The leukocyte gene signature matrix LM22 with 1,000 permutations was used to calculate the putative abundance of immune cells^24^. Data with "CIBERSORT" values of p < 0.05 were filtered and retained for subsequent analysis. We conducted Spearman's rank correlation analysis of the disease hub genes and immune cells to further explore the immune mechanism during the development of AD with MS. The results were visualized with the "corrplot" package.

### Metabolism-associated pathways analyses

The hallmark gene sets, containing 50 representative pathways, covering well-defined gene sets involving development, immunity, signaling pathways, etc., were downloaded from Molecular Signatures Database (http://www.gsea-msigdb.org/gsea/msigdb/, MSigDB-Hallmark version 7.4)^25^, as gene sets for ssGSEA analysis of AD and MS datasets^26^. Metabolism-associated pathways were picked out. Correlations between these metabolism-associated pathways and 9 hub genes were determined using Spearman correlation analysis to reveal AD and MS, respectively.

### Single-Cell RNA-Seq analysis of thoracic aortic aneurysms

We searched for a single-cell RNA-seq dataset of the thoracic aortic aneurysm to further validate our 9 hub genes. We downloaded single-cell RNA seq profiles (GSE213740) from the Gene Expression Omnibus (GEO) database, including 6 patients with AD and 3 controls. First of all, we carried out data quality control. We identified cells expressing more than 200 genes but no more than 6000 genes.

Meanwhile, 10% of mitochondrial genes and 3% of erythrocyte genes were selected as the threshold values for further filtering. After identifying 3,000 hypervariable genes for analysis, the number of principal components (PCs) was adjusted to 10 to generate cell clusters that were then exhibited and annotated using the "UMAP" diagram^27^. De-batching between different samples was performed using the method "Harmony". We next selected the top ten different expression genes in each cluster using the "FindAllmarkers" function from the 'Seurat' R Package^28^. Then 34 clusters in total were discovered.

We used previously published literature to annotate and cluster the cell clusters^29^. Between the expression profiles of each cell and those of the reference sample, Spearman's correlation was calculated. We utilized the "FindMarkers" method to identify genes that differed significantly between TAA and normal cells.

### Statistical analysis

All statistical analyses were conducted using R Studio (4.1.2). A student's t-test was used to compare AD and normal samples and MS and normal samples. ROC and Precision-Recall analyses were performed to estimate the discriminatory value of hub genes. Statistical significance was set at p < 0.05.

### Ethics statement

This article was from public data sets, and all data can be obtained from public websites.

## Results

### Identification and functional enrichment analysis of common DEGs

**Figure 2:**
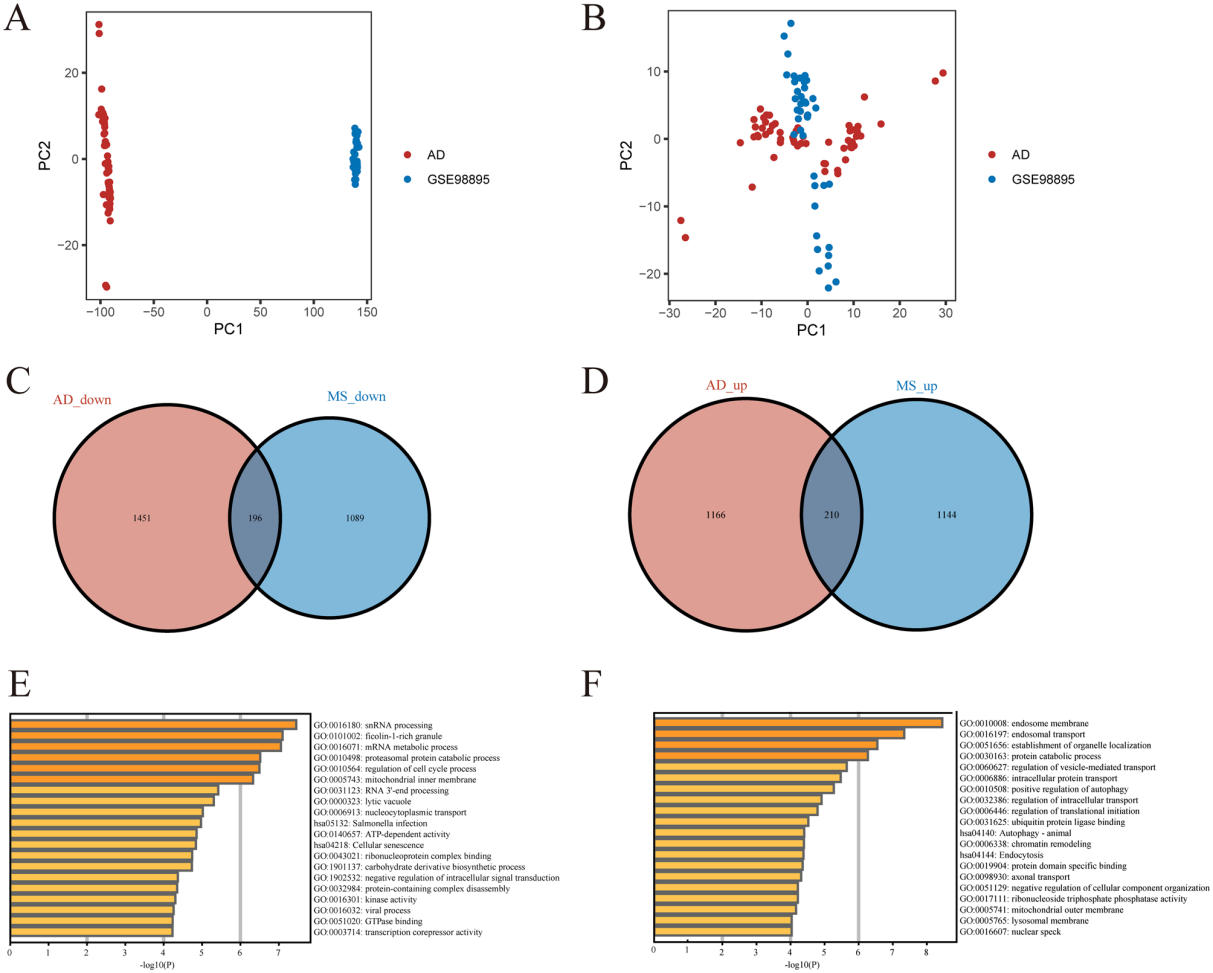
PCA of before and after elimination of batch effect and identification and functional enrichment analysis of common DEGs. (**A**) Represent the distribution of all samples from the AD combined dataset and GSE98895 dataset before elimination of batch effect. (**B**) Represent the distribution of all samples from the AD combined dataset and GSE98895 dataset after eliminating the batch effect. (**C,D**) A total of 210 common upregulated and 196 common down-regulated DEGs were identified after taking the intersection of DEGs in AD and MS. AD, aortic dissection; MS, metabolic syndrome. (**E**) Enrichment analysis of co-down-regulated genes of AD and MS. (**F**) Enrichment analysis of co-upregulated genes of AD and MS.

Batch effects had been eliminated with Rank-In in all samples from the AD combined dataset and GSE98895 dataset, as shown in Fig. 2A,B. The 3023 DEGs (1376 up- and 1647 down-regulated) were screened between AD and control subjects using the 'limma' package in Fig. 2C,D. There were 2639 DEGs (1354 up- and 1285 down-regulated) in MS patients compared to healthy controls. A total of 406 common DEGs (210 up- and 196 down-regulated) were identified after taking the intersection of the Venn diagrams. GO and KEGG Pathway enrichment analyses were performed to analyze the biological functions and pathways involved in AD and MS, respectively.

The down-regulated genes co-expressed by AD and MS were enriched in snRNA processing, ficolin-1-rich granule, mRNA metabolic process, proteasomal protein catabolic process, regulation of cell cycle process, mitochondrial inner membrane, RNA 3'-end processing, lytic vacuole, and nucleocytoplasmic transport (Fig. 2E).

In contrast, the common upregulated DEGs of AD and MS were significantly enriched in snRNA processing, ficolin-1-rich granule, mRNA metabolic process, proteasomal protein catabolic process, regulation of cell cycle process, and mitochondrial inner membrane (Fig. 2F).

### Weighted gene co-expression network analysis and key modules identification

**Figure 3:**
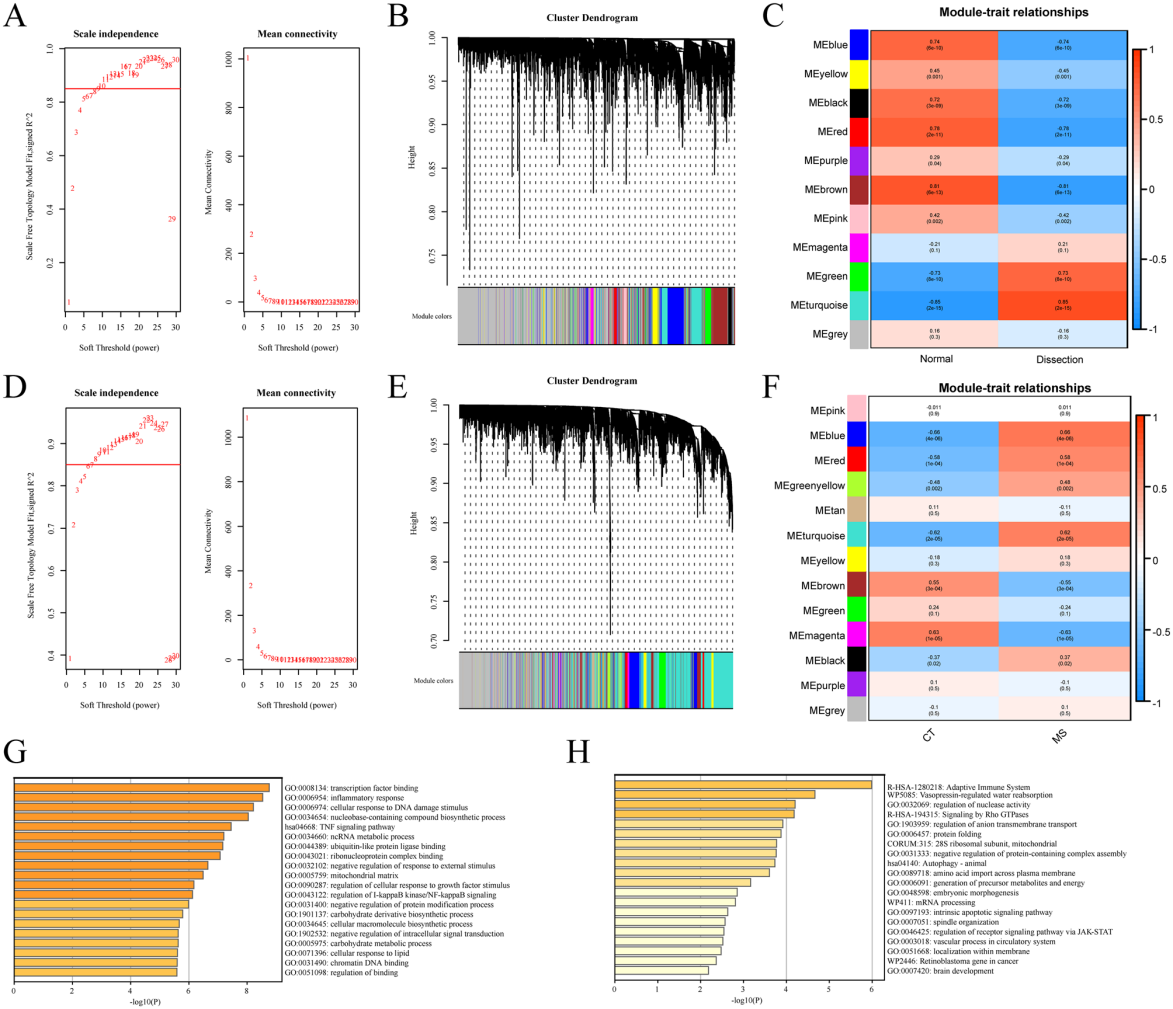
Construction of weighted co-expression network for the AD combined dataset and MS dataset, as well as enrichment analysis of key modules. (**A,D**) Network topology analysis of different soft threshold power. (**B,E**) Dendrograms of genes acquired by mean linkage hierarchical clustering. The allocation of modules decided by Dynamic Tree Cutting is displayed in the colored rows below the dendrogram. (**C,F**) Each column represents a clinical feature (AD or MS and control), and each row denotes an ME. The correlation coefficient and P-value are contained in each cell. ME: module eigengene. (**G,H**) GO and KEGG enrichment analysis of positively (**G**) and negatively (**H**) related key modules in AD and MS.

In the AD combined dataset and MS dataset, we used WGCNA to find co-expressed gene modules. The scale-free topological index was 0.85 when the soft thresholds for AD and MS were 9 and 7, respectively, as illustrated in Fig. 3A,D shows the derived gene dendrograms and their corresponding module colors.

The modules that were most relevant to the disease were identified. The heatmaps of the correlation between values of module eigengene and clinical features showed that the green (r = 0.73, P = 8E − 10) and turquoise (r = 0.85, P = 2E − 15) modules were significantly positively related with AD, while the blue (r = 0.74, P = 6E − 10), yellow (r = 0.45, P = 0.001), black (r = 0.72, P = 3E − 9), red (r = 0.78, P = 2E − 11), purple (r = 0.29, P = 0.04), brown (r = 0.81, P = 6E − 13) and pink (r = 0.42, P = 0.002) modules were significantly negatively related with AD. Similarly, the blue (r = 0.66, P = 4E − 6), red (r = 0.58, P = 1E − 4), green-yellow (r = 0.48, P = 0.002), turquoise (r = 0.62, P = 2E − 5), and black (r = 0.37, P = 0.02) modules were substantially positively related with MS, while the brown (r = 0.55, P = 3E − 4) and magenta (r = 0.63, P = 1E − 5) modules were substantially negatively related with MS (Fig. 3B,C,E,F).

To investigate the possible pathogenesis of AD and MS, enrichment analysis of their positively or negatively related key modules was performed, respectively. Function enrichment analysis showed that in AD and MS, the positively related modules were mainly associated with transcription factor binding, inflammatory response, TNF signaling pathway, and regulation of I-κB kinase/NF-κB signaling, etc. (Fig. 3G). Interestingly, we found that they were also mainly enriched in the cellular response to the lipid pathway, which was closely associated with the development and progression of AD and MS. Therefore, it may hint that a close link exists between AD and MS at the molecular level. Relatively, the negatively related modules were mainly associated with adaptive immune response, vasopressin-regulated water reabsorption, regulation of receptor signaling pathway via JAK-STAT, and vascular process in the circulatory system, etc. (Fig. 3H).

### Identification of hub genes of AD with MS via LASSO, random forest, and XGBoost algorithms

**Figure 4:**
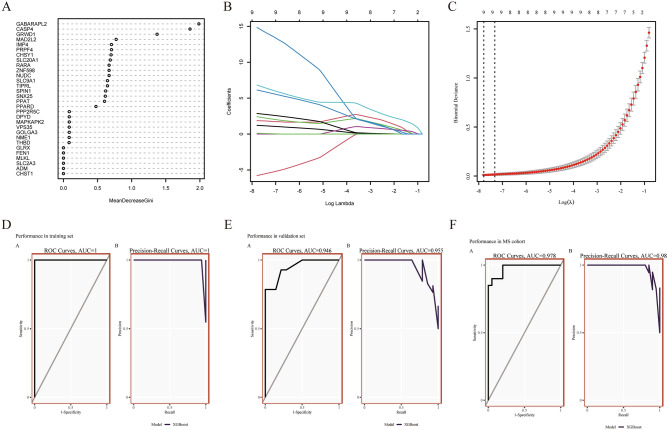
Machine learning in screening candidate diagnostic biomarkers (hub genes) for AD with MS and evaluating their diagnostic efficacy. (**a**) 30 hub gene candidates from the random forest classifier extracted through MeanDecreaseGini. (**b,c**) Biomarkers screening in the Lasso model. The number of genes (n = 9) corresponding to the lowest point of the curve is the most suitable for AD with MS diagnosis. (**d**) The ROC and Precision-Recall curves estimate the diagnostic performance of hub genes in the training set. (**e**) The ROC and Precision-Recall curves evaluate the diagnostic performance of hub genes in the validation set. (**f**) The ROC and Precision-Recall curves evaluate the diagnostic performance of hub genes in the MS cohort.

Two algorithms were applied for selecting hub genes of AD with MS. For the random forest algorithm, 30 hub gene candidates with MeanDecreaseGini > 0 were determined, including *CHST1, ADM, SLC2A3, MLKL, FEN1, GLRX, THBD, NME1, GOLGA3, VPS35, MAPKAPK2, DPYD, PPP2R5C, PPARD, PPAT, SNX25, SPIN1, TIPRL, SLC9A1, NUDC, ZNF598, RARA, SLC20A1, CHSY1, PRPF4, IMP4, MAD2L2, GRWD1, CASP4,* and *GABARAPL2* (Fig. 4A). Thus, we chose the minimum criteria for building the LASSO classifier due to higher accuracy by comparisons, and 12 hub gene candidates were identified (Figs. 4B,C). Following intersection, 9 hub genes shared by random forest and LASSO algorithms were finally identified (*CHSY1, DPYD, IMP4, MAD2L2, MAPKAPK2, PRPF4, SLC9A1, SLC20A1,* and *TIPRL*). We estimated the diagnostic performance of each hub gene in predicting AD in the combined GSE52093, GSE98770, and GSE147026 cohorts. The AUC values of ROC curves were 0.987 of *CHSY1*, 0.731 of *DPYD*, 0.971 of *IMP4*, 0.992 of *MAD2L2*, 0.987 of *MAPKAPK2*, 1.000 of *PRPF4*, 0.954 of *SLC9A1*, 1.000 of *SLC20A1*, and 0.924 of *TIPRL* (Supplementary Fig. 1A–I), demonstrating that these hub genes enabled to diagnose AD.

The diagnostic model was established through the XGBoost algorithm using AD datasets of GSE52093, GSE98770, and GSE147026 as training sets. When all 9 hub genes were fitted into one variable, the AUC value of both ROC and Precision-Recall curves was 1, demonstrating the favorable diagnostic efficiency in predicting AD (Fig. 4D).

The 9 hub genes were further verified in external datasets (the combined GSE153434 and GSE190635 datasets) and the MS dataset. In AD external datasets, the AUC value of ROC curves was 0.946, while the AUC value of Precision-Recall Curves was 0.955, indicating their potential in diagnosing AD (Fig. 4E). Moreover, in the MS dataset, the AUC value of ROC curves was 0.978, while the AUC value of Precision-Recall Curves was 0.98, demonstrating that they are capable of diagnosing MS. Hence, the hub genes possessed excellent diagnostic performance in predicting AD with MS (Fig. 4F).

### Immune cell infiltration analysis

**Figure 5:**
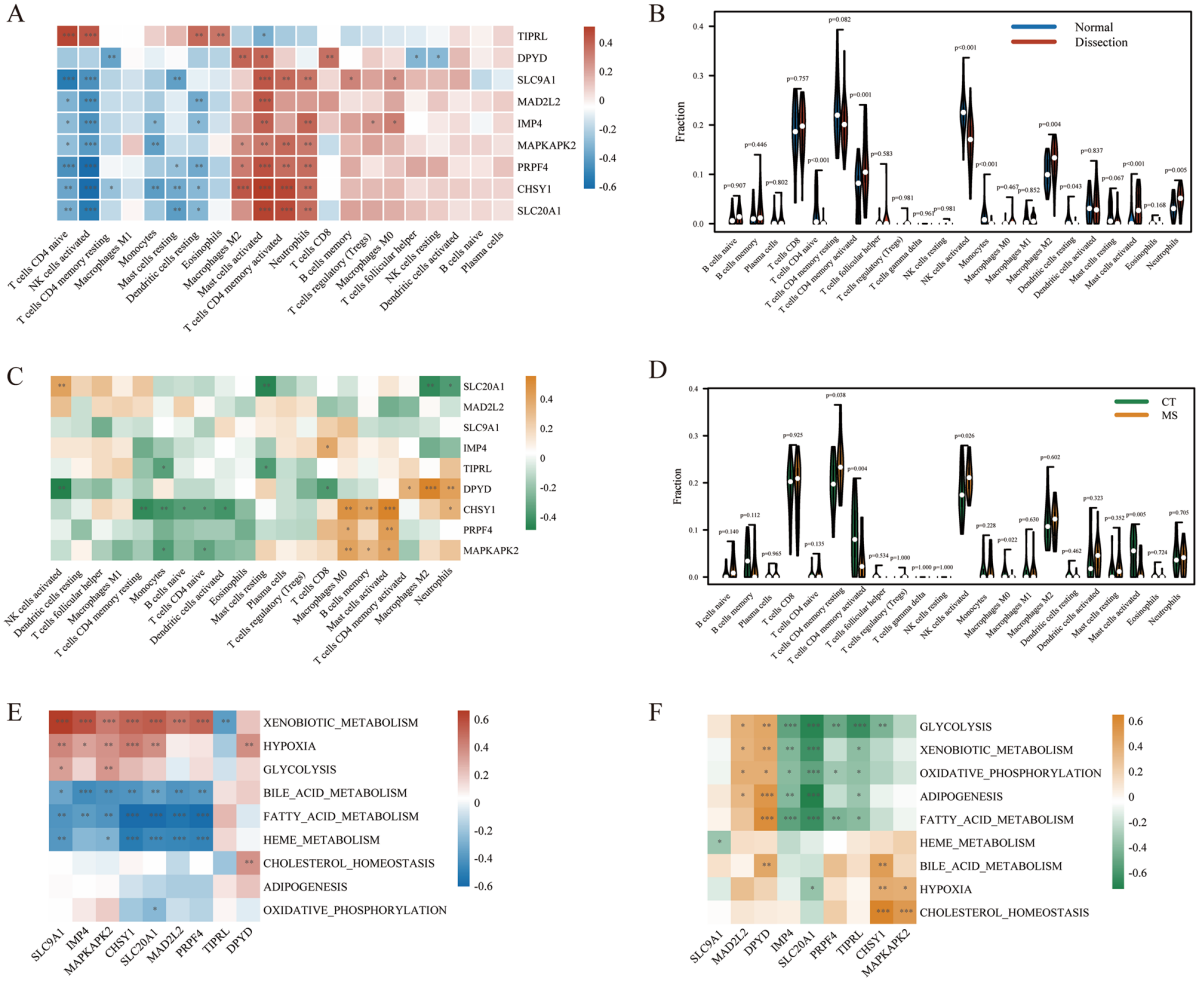
Immune cell infiltration and metabolism-associated pathways analyses in AD and MS. (**A**) Analysis of correlation between hub genes and immune cells in AD. (**B**) The violin graph shows the difference in immune infiltration between AD and control samples. The control samples are shown in blue and AD samples in red. (**C**) Analysis of correlation between hub genes and immune cells in MS. (**D**) The violin graph shows the difference in immune infiltration between MS and control samples. The control samples are shown in green, and the MS samples are shown in orange. (**E**) Analysis of correlation between hub genes and metabolism-associated pathways in AD dataset. (**F**) Analysis of correlation between hub genes and metabolism-associated pathways in MS dataset. *p < 0.05, **p < 0.01, ***p < 0.001. Normal, normal samples. Dissection, aortic dissection samples. *CT* control samples, *MS* metabolic syndrome samples.

Spearman's rank correlation analysis explored the correlation between the hub genes and different immune cells. In AD samples, except for *TIPRL*, the other genes had a positive correlation with activated mast cells, activated CD4 memory T cells, M2 macrophages, and neutrophils and a negative correlation with activated NK cells, naïve CD4 T cells, resting mast cells, and monocytes. *TIPRL* had a positive correlation with naïve CD4 T cells, activated NK cells, resting dendritic cells, and eosinophils and a negative correlation with activated mast cells (Fig. 5A). Regarding immune cell infiltration, compared to control groups, the violin plot demonstrated that AD patients had a higher level of activated CD4 memory T cells (P = 0.001), M2 macrophages (P = 0.004), activated mast cells (P < 0.001), and neutrophils (P = 0.005) and a lower level of naive CD4 T cells (P < 0.001), activated NK cells (P < 0.001), monocytes (P < 0.001) and resting dendritic cells (P = 0.043) (Fig. 5B).

In MS samples, *CHSY1* had a remarkable positive correlation with activated mast cells, M0 macrophages, and memory B cells. In contrast, there was a remarkable negative correlation between *CHSY1* and resting CD4 memory T cells, monocytes, naïve B cells, naïve CD4 T cells, and activated dendritic cells. The *DPYD* had a significant positive correlation with M2 macrophages, neutrophils, and activated CD4 memory T cells. At the same time, there was a significant negative correlation between *DPYD* and both activated NK cells and CD8 T cells. *MAPKAPK2* had a positive correlation with M0 macrophages, activated mast cells, and memory B cells and a negative correlation with monocytes and naïve CD4 T cells. *SLC20A1* positively correlated with activated NK cells and negatively correlated with resting mast cells, M2 macrophages, and neutrophils. In addition, *PRPF4* had a positive correlation with activated mast cells and M0 macrophages, while *IMP4* only had a positive correlation with CD8 T cells. Both of them didn't have any negative correlation with infiltrated immune cells. Whereas the statistical analysis showed, TIPRL only had a negative correlation with resting mast cells and monocytes but not any significant positive correlation with various infiltrated immune cells (Fig. 5C).

For MS vs. control groups, MS patients had higher resting CD4 memory T cells (P = 0.038). They activated NK cells (P = 0.026) and a lower level of activated CD4 memory T cells (P = 0.004), M0 macrophages (P = 0.022), and activated mast cells (P = 0.005) (Fig. 5D).

### Metabolism-associated pathways analyses

The metabolism-associated signatures picked out included Xenobiotic metabolism, Hypoxia, Glycolysis, Bile acid metabolism, Fatty acid metabolism, Heme metabolism, Cholesterol homeostasis, Adipogenesis, And Oxidative phosphorylation pathways. To explore potential metabolism-associated pathways in the 9 hub genes involved, the correlation between 9 hub genes and metabolism-associated pathways was analyzed. As shown in Fig. 5E, in the AD dataset, the *SLC9A1, IMP4, MAPKAPK2, CHSY1, SLC20A1, MAD2L2,* and *PRPF4* had a significant positive association with the Xenobiotic metabolism pathway, while TIPRL had a significant negative association with it. The *SLC9A1, IMP4, MAPKAPK2, CHSY1, SLC20A1,* and *DPYD* had a remarkable positive association with the HYPOXIA pathway. Besides, *SLC9A1* and *MAPKAPK2* also had a remarkable positive association with the Glycolysis pathway. Specifically, a significant negative association was observed between *SLC9A1, IMP4, MAPKAPK2, CHSY1, SLC20A1, MAD2L2,* and *PRPF4* genes (except for *TIPRL* and *DPYD*) and pathways including Fatty acid metabolism, Bile acid metabolism, and Heme metabolism, especially Fatty acid metabolism pathway. In the MS dataset, *MAD2L2* and *DPYD* were found to be positively correlated with Glycolysis, Xenobiotic metabolism, Oxidative phosphorylation, Adipogenesis, and Fatty acid metabolism pathways. At the same time, the *IMP4, SLC20A1, PRPF4,* and *TIPRL* were almost negatively correlated with these five pathways. In addition, the *CHSY1* and *MAPKAPK2 are* positively associated with pathways including Cholesterol homeostasis, Hypoxia, and Bile acid metabolism (Fig. 5F).

### Validation of hub genes in single-cell RNA-Seq data

**Figure 6:**
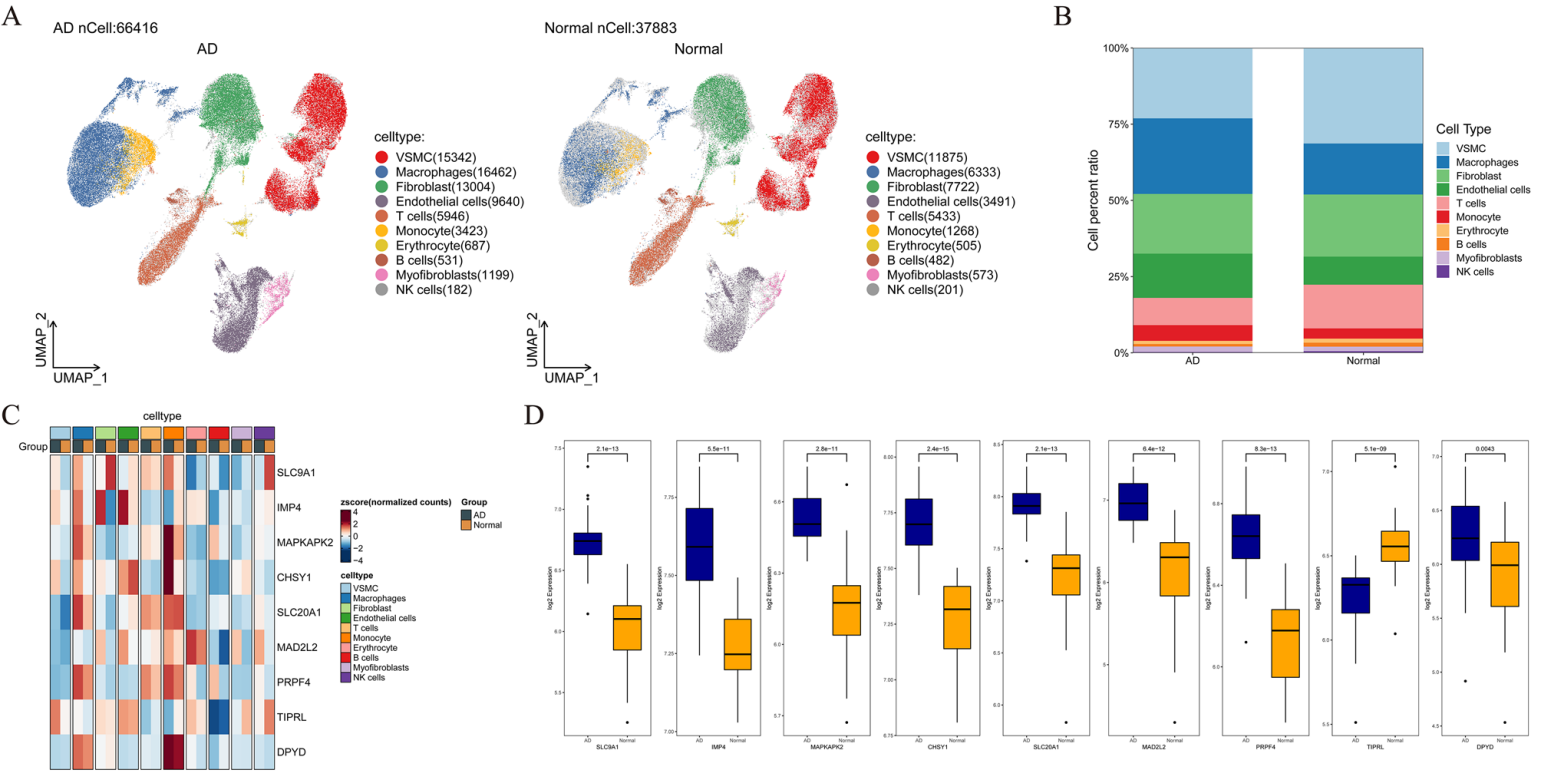
Characterization of scRNA-seq from AD. (**A**) UMAP showed the visualization of cell subsets in normal samples and AD tissues. (**B**) The cell proportion showed the difference of cell subsets between the two groups. (**C**) Heat map showing the difference in expression of hub genes in different cell subpopulations in AD and normal patients. (**D**) Gene expression of hub gene in normal samples and AD patients. Acute type A aortic dissection: ATAAD.

On the single-cell dataset, a quality control procedure was performed. As shown in Supplementary Fig. 2A–C. UMAP visualization of different patients after batch removal by Harmony (Supplementary Fig. 2D). As in Supplementary Fig. 3A–C, for the annotation of cell subpopulations, we used different signature cell makers for annotation. In the end, the single-cell sample was divided into 10 cell subsets with a total of 104,299 cells (Supplementary Fig. 3D–F). As shown in Fig. 6A, the tissue microenvironment of patients with AD is divided into 8 cell subsets, roughly divided into VSMC, Macrophages, Fibroblast, Endothelial cells, T cells, Monocyte, Erythrocyte, B cells, Myofibroblasts and NK cells. Among them, we found that the proportion of inflammatory and immune-related cells (including macrophages, Endothelial cells and monocytes) in TAA patients was higher than that in normal controls (Fig. 6B). Therefore, it was also proved that the occurrence and development of AD was closely related to inflammatory and immune response. Then, we used heat maps to visualize the expression levels of 9 hub genes in two cell samples and ten different cell types. Compared with the normal group, the expression levels of 9 hub genes in AD group were increased (Fig. 6C). Compared with normal tissues, except for the low expression of gene PRPF4 in AD tissues, the other genes were highly expressed in AD tissues (p < 0.05) (Fig. 6D).

**Figure 7:**
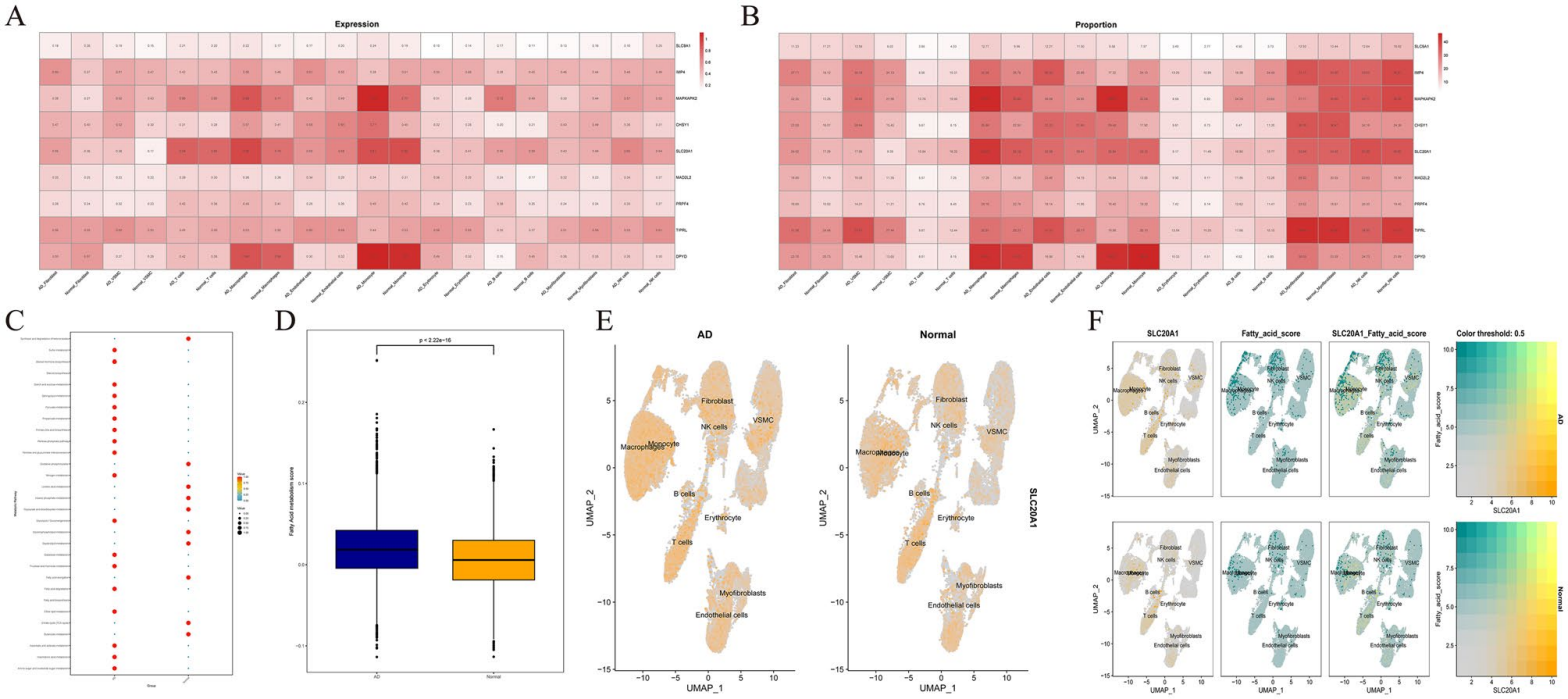
Expression of fatty acid metabolism signal pathway and key genes in TAA patients. (**A**) The correlation heatmap showed the expression quantities of 9 hub genes in different cell types from TAA and normal cell samples. (**B**) The correlation heatmap showed the expression proportion of 9 hub genes in different cell types from TAA and normal cell samples. (**C**) GSEA enrichment analysis of enrichment signal pathways in patients with AD and normal patients. (**D**) The box chart shows the expression of Fatty Acid metabolism score in patients with AD and normal patients. (**E**) UMAP shows the expression of SLC20A1 in different cells of normal and AD tissues. (**F**) UMAP shows the co-localized expression of SLC20A1 and fatty acid metabolism in normal and TAA tissues. Type A aortic *TAA*.

In addition, important correlation heatmaps (including expression quantities and proportional correlation heatmap) between nine hub genes and different cell samples and cell types were drawn to present these hub genes' cytological localization and expression differences (Fig. 7A,B). Enrichment of metabolic signal pathways between AD and normal patients showed that multiple fatty acid pathways were enriched in AD patients (Fig. 7C). Our results demonstrated that fatty acid metabolic pathways are differentially expressed in endothelial cells of normal and AD patients (Fig. 7D). SLC20A1 gene expression was highly expressed in cells of multiple AD patients compared to normal patient tissues (Fig. 7E). As shown in Fig. 7F, the gene SLC20A1 co-localizes with fatty acid metabolic pathways in AD patients and normal humans in a variety of cells.

## Discussion

The regulation of aortic structure and function involves interactions and highly coordinated actions between the aortic cell spectrum and the ECM (extracellular matrix)^30–38^. Several pathways that tightly control aortic cells have been identified and play a key role in regulating aortic structure and function^39,40^. Interestingly, these pathways control aortic cells in a cell type-specific manner. For example, the same pathways can signal cell-specific behaviors that lead to distinct or even opposing outcomes in terms of aortic protection and destruction. The complexity of aortic wall signaling poses a challenge for the development of diagnostic and therapeutic strategies for aneurysms and dissections (AAD). There also have been no previous studies that have combined the two diseases.

Furthermore, machine learning methods and model generation have not been used in diagnosing AD. Therefore, this study used a series of integrated bioinformatics analysis and machine learning methods to construct a diagnostic model of AD in MS patients and to assess the diagnostic value of AD in MS patients. The most noteworthy discovery is that we identified nine essential hub candidate genes (*MAD2L2, IMP4, PRPF4, CHSY1, SLC20A1, SLC9A1, TIPRL, DPYD,* and *MAPKAPK2*) and developed a diagnostic model for diagnosing AD in MS patients. They showed the most remarkable correlation with the fatty acid metabolism pathway. ScRNA-seq data analysis further indicates a prominently increased expression of all hub genes in smooth muscle cells, monocytes, and T cells. Besides, the *SLC20A1*, *CHSY1,* and *TIPRL* also had a significantly high expression level in endothelial cells. We, therefore, conclude these hub genes might play essential roles in the fatty acid metabolism pathway and smooth muscle and endothelial cell dysfunction during the progression of AD and MS.

SMC is the major cellular component of the aortic wall and plays a central role in maintaining aortic function and endostasis^41^. CHSY1 is a member of the chondroitin N -acetylgalactosaminyltransferase family, which plays a key role in the biosynthesis of chondroitin sulfate, a glycosaminoglycan (GAG) that attaches to the core protein to form chondroitin sulfate proteoglycan (CSPG)^42^. According to a report, TGF-β1 has been reported to cause phosphorylation of the transcription factor Smad2 linkage region through its receptor action, a response that is associated with TGF-β1-mediated mRNA expression of the GAG synthases CHSY1 and CHST11, two enzymes closely associated with GAG chain elongation in human VSMCs. These findings provide a better understanding of the signaling pathway controlling the length of proteoglycan GAG that promotes lipoprotein binding in the vessel wall and the development of atherosclerosis^43^. Furthermore, endothelin-1 signaling via ETB receptor (endothelin-1 type B receptor) utilizes cytoskeletal rearrangements and Rho (ROCK) kinase, but not map, leading to transactivation signaling of TβRI (transforming growth factor beta type I receptor) and phosphorylation of Smad2C, and increases CHSY-1 levels via this pathway^44^. In addition, a recent study on rat retinal microvascular endothelial cells (rrmec) showed that hyperglycemia in rrmec leads to significantly elevated mRNA levels of GAG biosynthetic enzymes (including extl -1,2,3, ext -1,2, chsy -1,3, and has2,3), and that these elevations may be a compensatory response to overall glycocalyx loss^45^.

Tip41-like protein (TIPRL) is an evolutionarily conserved pp2a family phosphatase-binding protein that is a negative regulator of protein phosphatase 4 (PP4). It inhibits PP4 activity, allowing H2AX (histone H2A variant) phosphorylation and subsequent DNA damage response^46^. However, few studies directly analyze the role of TIPRL in AD, or MS. Most studies have focused on digestive system tumors and lung cancers. For example, it has been shown that the TOR signaling pathway regulatory -like (TIPRL) is highly upregulated in hepatocellular carcinoma (HCC) cells, inhibiting the TRAIL-induced apoptotic cascade by forming the MKK7/PP2Ac/TIPRL complex and blocking JNK phosphorylation, thereby promoting TRAIL (tumor necrosis factor-related apoptosis-induced ligand) resistance^47^. In non-small cell lung cancer (NSCLC), upregulation of TIPRL enhances the autophagic activity and enables autophagy to clear metabolic and cellular stress, conferring a survival advantage to cancer cells^48^. In contrast, in atypical TGF-β pathways, activated TGF-β receptor complexes signal through several pathways, such as TAK1 (TGF-β-activated kinase 1), p38 MAPK (p38 mitogen-activated protein kinase), ERK (extracellular signal-regulated kinase), JNK (Jun N-terminal kinase), and NF-κB (nuclear factor-κB), promoting aortic disruption and the development of AAD^10^. Therefore, we believe there is a deep relationship between TIPRL, aortic diseases, and metabolic disorders, which emphasizes its importance in future research.

The sodium-dependent phosphate transporter protein SLC20A1 is required for inorganic phosphate (Pi)-elevation-induced matrix mineralization and phenotypic transdifferentiation in vascular smooth muscle cells (VSMC). Previous studies identified the Rap1 guanine nucleotide exchange factor (RapGEF1) as a slc20a1-interacting protein, and RapGEF1 promotes ERK1/2 phosphorylation through Rap1 activation^49^. In aortic disease, SMC may exhibit a phenotypic switch, resembling mesenchymal stem cells, myofibroblasts, fibroblasts, osteoblasts, chondrocytes, macrophage-like inflammatory cells, foam cells, or adipocyte-like cells^50–53^. This phenotypic switch of SMCs is often observed in the aortas of patients with either heritable or sporadic AAD^54–56^. The most common phenotypic changes observed in disseminated TAAD or AAA patients are a decrease in SMC protein expression (e.g. SM22-α) and an increase in inflammatory protein expression (e.g. matrix metalloproteinase-2 and MMP-9)^32^. Fatty acid metabolic pathways were also seen to be enriched in a variety of immune cells in our single cell analysis, particularly in endothelial cells, which may suggest to us that these metabolic pathways may be involved in the progression of AD disease.

Moreover, PiT1-deficient mice were protected against high-fat-diet-induced obesity and diabetes^57^. For the fatty acid metabolic pathway, a study of ruminants shows they have a unique utilization of phosphate (Pi) based on the so-called endogenous Pi recycling to guarantee adequate Pi supply for ruminal microbial growth and for buffering short-chain fatty acids. Large amounts of Pi enter the gastrointestinal tract by salivary secretion. The high saliva Pi concentrations are generated by active secretion of Pi from the blood into primary saliva via basolateral sodium (Na +)-dependent Pi transporter type II. The subsequent intestinal absorption of Pi is mainly carried out in the jejunum by the apical located secondary active Na + -dependent Pi transporters Napi IIb (SLC34A2) and PiT1 (SLC20A1)^58^. Moreover, in PiT1-deficient mice, specific knockout of Pit1 in hepatocytes significantly improved glucose tolerance and insulin sensitivity, enhanced insulin signaling, and decreased hepatic lipogenesis^57^. In another microarray-based gene expression analysis of murine brown and subcutaneous adipose tissue, SLC20A1 screened out as a critical gene was further validated using quantitative RT-PCR that had a relatively low expression in subcutaneous white adipose tissues^59^. SLC20A1 may be an important gene involved in the occurrence and development of AD.

Our study had several limitations. First, although we pooled five AD datasets, the samples remained few, and the value of the diagnostic model was relatively high due to the limited sample size. The results should be subsequently confirmed in a more large-scale study with a large sample size and laboratory experiments. Second, several hub genes are mainly expressed in SMCs and endothelial cells, which have a special relationship with fatty acid metabolism. However, crosstalk in these genes, the interaction between them and dysregulated cells, and pathways were still worth investigating.

## Conclusion

Our study systematically discovered nine candidate hub genes (*MAD2L2, IMP4, PRPF4, CHSY1, SLC20A1, SLC9A1, TIPRL, DPYD,* and *MAPKAPK2*) and provided the diagnostic model for diagnosing AD with MS by various bioinformatics analysis and machine learning algorithms. We also point out the dysregulated immune cell proportion and metabolism-associated pathways in AD with MS. Our study could provide potential new insights for the further study of the molecular mechanism of AD with MS.

### Supplementary Information

Supplementary Figures.

## Data Availability

The original contributions presented in the study were included in the article, further inquiries can be directed to the corresponding author.
