# Supplementary material for: Identification of co-diagnostic effect genes for aortic dissection and metabolic syndrome by multiple machine learning algorithms
Source: Sci Rep. 2023 Sep 8;13:14794. doi: 10.1038/s41598-023-41017-4 (PMC10491590; doi:10.1038/s41598-023-41017-4)
Supplement: Supplementary file 1 — Supplementary Figures. [file 41598_2023_41017_MOESM1_ESM.docx]

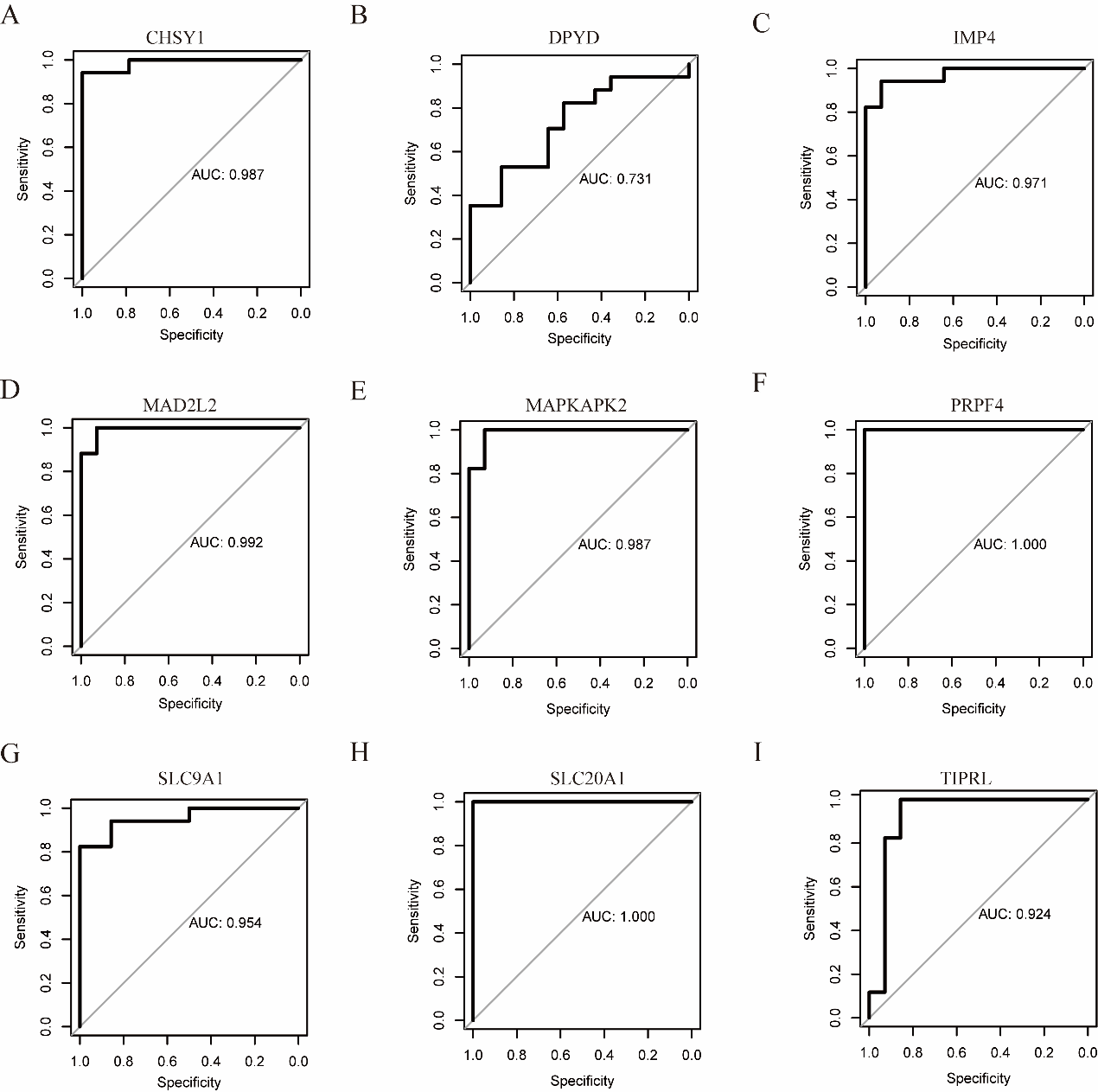


**Supplementary Figure** 1：Receiver operating characteristic curve of diagnostic genes(A-I).


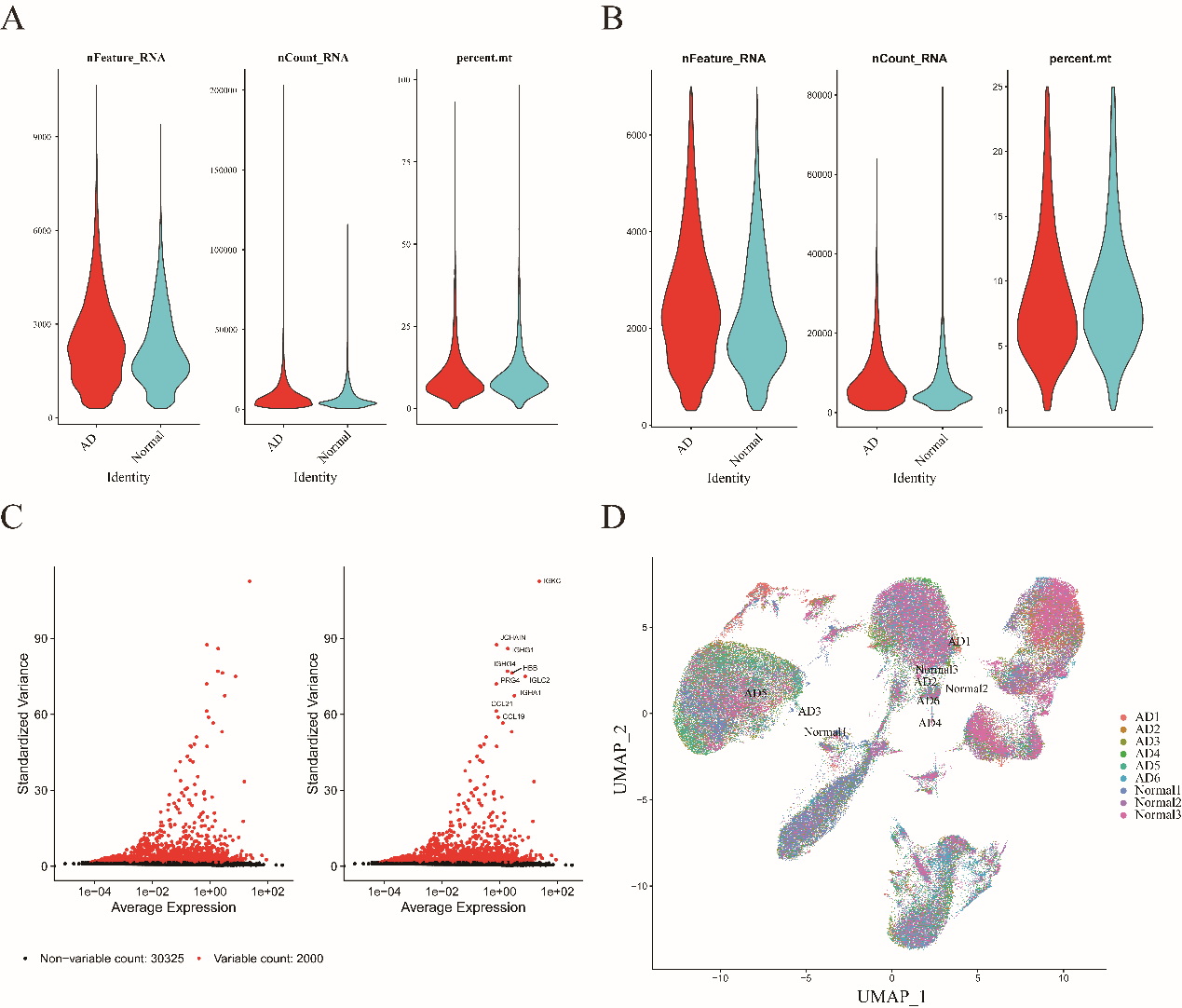


**Supplementary Figure** 2：Quality control process of single-cell. (A-C) The number of cells, the proportion of mitochondria, proportion of red blood cells before and after quality control. (D)

UMAP demonstrated the distribution of cells in AD and normal patients after performing harmony.


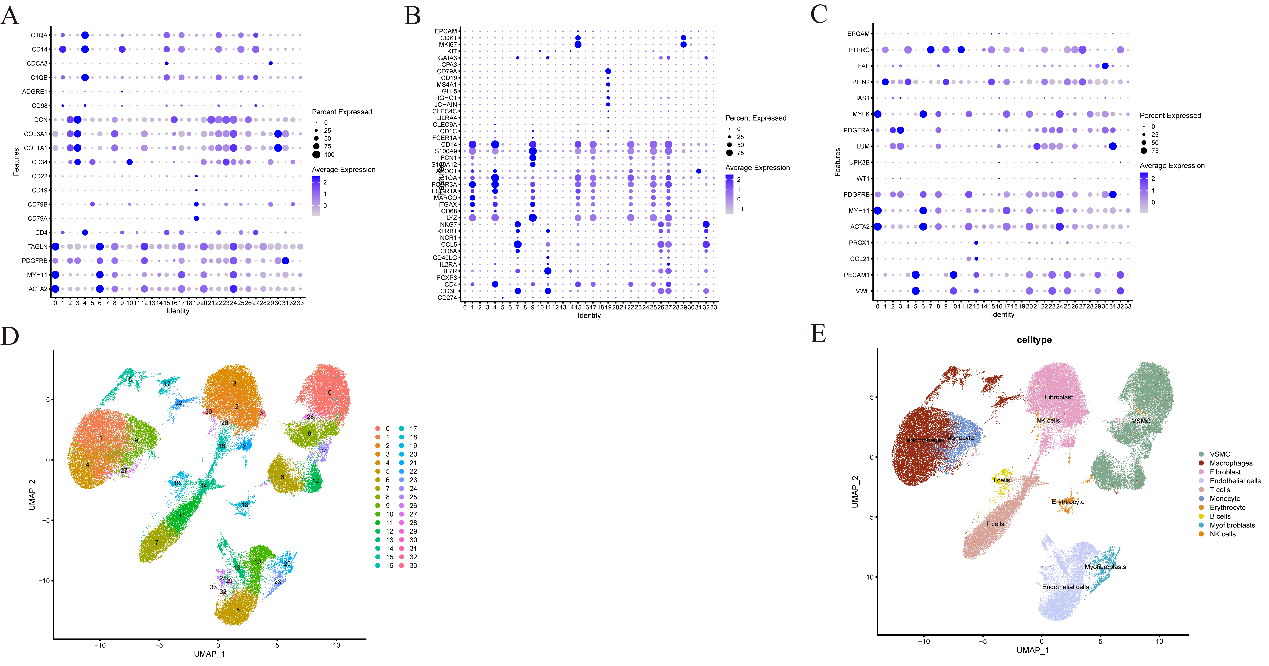


**Supplementary Figure** 3：Single-cell subpopulation subgroups of AD patients. (A-C) Heat map showing the expression of maker genes annotated in different cell fractions. (D) UMAP demonstrated cell fractionation before performing annotation. (D) UMAP demonstrated the fractional population composition of cells after annotation was performed.
